# Assessing the practice of total neoadjuvant therapy for rectal cancer: an online survey among radiation oncology departments in Germany and German-speaking regions of Austria and Switzerland

**DOI:** 10.1007/s10238-024-01495-w

**Published:** 2024-10-19

**Authors:** Stefan Knippen, Guido Hildebrandt, Florian Putz, Lasse Leon Gossé, Jörg-Peter Ritz, Marciana-Nona Duma

**Affiliations:** 1 Department of Radiation Oncology, Helios Clinics of Schwerin-University Campus of MSH Medical School Hamburg, Schwerin, Germany; 2 Department of Radiation Oncology, University Medical Center Rostock, Rostock, Germany; 3 Department of Radiation Oncology, University Medical Center Erlangen, Friedrich-Alexander-Universität Erlangen-Nürnberg, Erlangen, Germany; 4 Department of General and Visceral Surgery, Helios Clinics of Schwerin-University Campus of MSH Medical School Hamburg, Schwerin, Germany; 5 Department for Human Medicine, MSH Medical School Hamburg, Hamburg, Germany

**Keywords:** TNT, Survey, Rectal cancer, Radiation, Online, Watchfull waiting

## Abstract

Total neoadjuvant therapy (TNT) of rectal cancer improves rates of pathological complete remission and progression-free survival. With improved clinical response rates, interest grew in a non-operative approach/watch and wait (WaW) for this disease. In 2020, the working groups of ACO/AIO/ARO published a consensus statement on the use of TNT, including a non-operative approach. However, the best combination scheme remains unclear. Despite the increasing use of TNT, there is a lack of comprehensive data on its current implementation and practices. To address this knowledge gap, a multicenter survey was conducted to capture the use of TNT protocols in German-speaking radiotherapy departments. At the beginning of 2023, a GDPR-compliant online survey was conducted in Germany, Austria, and German-speaking Switzerland. The questionnaire comprised 43 questions covering various aspects of TNT, including chemotherapy and WaW concepts. Most respondents (98.4%) confirmed awareness of the consensus on TNT for rectal cancer. Institutions treated an average of 30.22 rectal cancer patients annually. Most respondents (76.2%) reported treating over 80% of patients neoadjuvantly. Regarding TNT, 33.3% treated 21–50% with such a protocol. No significant association was found between the institution type and TNT application. In 62/63 cases, tumor board discussion was standard before offering TNT. VMAT was the predominant technique (82.5%). For rectal cancer dosing, the 50/50.4Gy scheme was most common, followed by 45Gy with a boost and the 5 × 5Gy scheme. Dosing schemes for TNT varied slightly, with more participants reporting the use of 5 × 5Gy compared to radiation therapy for rectal cancer in general. CBCT was the primary IGRT method (88.9%). Larger hospitals typically administered chemotherapy themselves, while private practices collaborated with medical oncologists (*p* < 0.0001). The most common concurrent chemotherapy drugs were 5-fluorouracil/capecitabine (64.4%) and oxaliplatin (37.3%). A WaW strategy was reported to be institutional implemented by 63.8%. The timing of offering WaW was split, with 50% offering it after radiochemotherapy and 47% during the informed consent talk. For planned WaW, 62% prefer normofractionated TNT. TNT appears to be widely implemented in the German-speaking radio-oncological community, regardless of the type of institution. Image-guided therapy, multidisciplinary team decisions, and internal guidelines play an important role. TNT seems to have already altered treatment protocols for rectal cancer toward an organ-preserving approach in selected cases. In these WaW cases, normofractionation appears to be preferred over hypofractionation.

**Supplementary Information:**

The online version contains supplementary material available at 10.1007/s10238-024-01495-w.

## Introduction

Recent trials, besides others the RAPIDO, PRODIGE 23 and OPRA study, have shown that intensified preoperative chemoradiotherapy is beneficial in the treatment of rectal cancer, improving pathological complete response rates and progression-free survival. These trials employed different combinations of chemotherapy, including induction chemotherapy followed by radiochemotherapy and 5 × 5Gy radiation followed by FOLFOX [1–4]. As clinical response rates have improved, there has been increasing interest in a non-operative approach in cases of clinical complete response in selected cases [4, 5]. In 2020, the working groups of surgical oncology, medical oncology, and radiation oncology (ACO/AIO/ARO) in Germany issued a consensus statement on the use of total neoadjuvant therapy (TNT) for rectal cancer, including a non-operative approach [6, 7]. However, the best combination scheme for achieving complete response rates remains unclear, and the choice of scheme is left to the treating physician. Despite its increasing adoption, there is a lack of comprehensive data on the current implementation and practices of TNT. Real-world practice has seen various adaptations of TNT schemes [8]. To address this knowledge gap, a multicenter survey was conducted to evaluate the utilization of TNT protocols in radiation oncology departments in German-speaking countries.

## Materials and methods

### Design of the survey

The aim of the survey was to collect data on the utilization of neoadjuvant therapy for rectal cancer, specifically focusing on total neoadjuvant therapy (TNT) regimens and treatment planning techniques employed. Participation was sought from radiation oncology departments in Germany, Austria, and German-speaking Switzerland. To ensure confidentiality and data privacy in accordance with German DSGVO (German Data Protection Regulation, GDPR), the survey was conducted using the secure online platform Unipark^®^, licensed from Medical School Hamburg and developed by Tivian XI^®^ GmbH in Cologne, Germany. The survey questionnaire consisted of 43 questions organized into six sections, each starting with a brief introductory page. These sections covered various aspects of TNT for rectal cancer, including general information on institutional characteristics, radiation therapy in the context of TNT, radiation therapy planning considerations, chemotherapy for rectal cancer, “watch and wait” concepts, and anonymous information related to the respondents' personal background. The complete survey can be found in the supplementary material. The survey link was distributed via email to publicly available contact addresses. The invitation email also included a QR code for respondents who preferred to answer the survey on their mobile devices. Prior to beginning the survey, participants were asked for their agreement to complete it. One Austrian clinic network required an additional internal review process, which was completed. Additionally, the email contact was requested to either answer the survey themselves or forward it to a physician involved in the field. Data analysis was conducted in IBM^®^ SPSS^®^ Statistics Version 26 and Microsoft Excel (Version 1808, Microsoft Corporation, Redmond, Washington, USA) using appropriate statistical methods, including descriptive statistics and inferential analyses where applicable. The survey was available for responses from January 22 to April 15, 2023, during which time one invitation email and one reminder email were sent to encourage participation. The results of the survey are presented comprehensively, providing insights into the current practices and landscape of TNT implementation in daily clinical practice. Ethical approval for this project was obtained from the ethics committee of the University Medicine Rostock with the reference number A 2022–0183. Adherence to the recommendations of the CHERRIES Checklist for Reporting Results of Internet E-Surveys by Eysenbach was done, whenever possible [9].

## Results

For Germany, a total of 361 publicly available email addresses of radiation oncology institutes registered at the DEGRO homepage were included in the study. Out of these, mail delivery failed in eight cases, with two of those cases being due to non-existent domains. However, working email addresses were found via internet search in three of these cases. In the case of Austria, registered email addresses from www.oegro-rt.at were utilized. Additionally, contact addresses to the head of department of one clinic network were obtained through a personal request after an internal review process for this survey, resulting in 16 additional contacts. For Switzerland, contact email addresses were obtained from www.sasro.ch, resulting in a total of 20 contact addresses. In total, 95 centers accessed the survey through the provided link or QR code, with only two centers (2.11%) refusing consent. Ten participants gave their informed consent but did not start the survey, resulting in a net participation of 83 participants. Out of these 83 participants, 63 were included in the analysis as they provided responses beyond general information.

### General information

In the first section, participants were asked to provide information about their country of work, institutional sponsorship, staffing and equipment, as well as the number of patients treated per year. Out of the respondents, 23 (36.5%) identified themselves as working in university hospitals or tertiary care hospitals, 27 (42.9%) as doctors in private practice or medical service centers, 12 (19%) as general hospitals, and one participant described their institution as a "regional oncology center" in a text field. The sponsorship of the institutions was classified as public in 26 cases (41.3%), private in 30 cases (47.6%), and church-funded in 7 cases (11.1%). The survey participants were predominantly from Germany, accounting for 81% (*n* = 51), followed by Austria with 7.9% (*n* = 5), and Switzerland with 11.1% (*n* = 7). Further questions focused on the number of radiation oncology specialists, linear accelerators, and medical physicist experts. Please refer to Table 1 for the detailed results, and Table 4 in the Supplement for details grouped by country. Two questions required free-text answers, which were the number of radiographers employed by the participating institutions (median 14, range 4–60; 95% CI 11.6–16.4) and the total number of patients treated (median 1125, 350–4000; 95% CI 1160–1516). This corresponds to an estimated annual total of approximately 64.600 treated patients in total.

**Table 1:** General information

Number of linear accelerators	*n* =	%
1–2	38	60.3
3–4	18	28.6
5	5	7.9
> 5	2	3.2
Total	63	
Number of radiation oncologists		
Up to 3	20	31.7
4–5	20	31.7
6–10	16	25.4
> 10	7	11.2
Total	63	
Number of MPE		
Up to 3	13	20.6
4–5	18	28.6
6–10	24	38.1
> 10	8	12.7
Total	63	

*MPE* Medical Physicist Expert

### Specific information

#### Questions addressing general topics of TNT

**Fig. 1:**
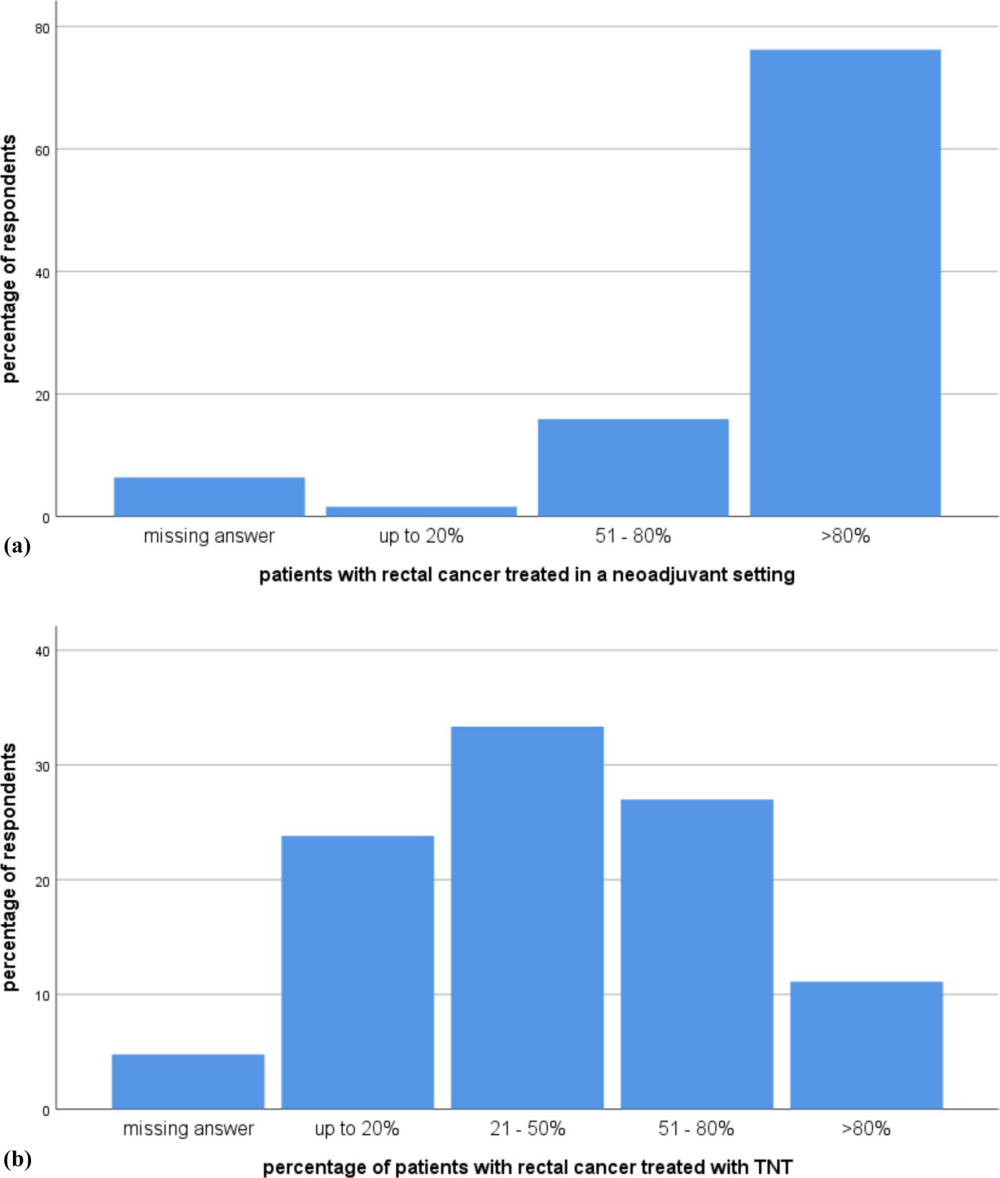
**a** Distribution of patients treated with neoadjuvant radio(chemo)therapy **b** Distribution of patients treated with neoadjuvant TNT

**Fig. 2:**
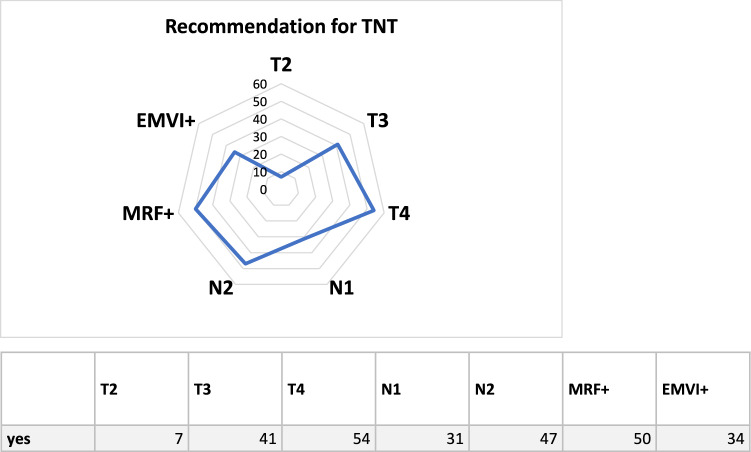
Radar Chart of quoted indication for TNT

The chapter started by asking participants if they are aware of the consensus opinion on the use of TNT for rectal cancer [7], with 62 (98.4%) responding "yes". The institutions represented by the participants treat an average of 30.22 rectal cancer patients per year (median 25, interquartile range 20–40, range 2–100, 95% CI 25.5–34.5). As expected, the majority of respondents reported treating a large portion of their rectal cancer patients with neoadjuvant therapy, with 76.2% treating > 80% of the patients neoadjuvant, 15.9% treating 51–80% of the patients neoadjuvant, and only one respondent treating less than 20% neoadjuvant (*n* = 4 missing data). TNT treatment demonstrated a different distribution, with 33.3% of participants treating 21–50% of their cases with TNT. The distribution of neoadjuvant radiochemotherapy and TNT application is shown in Fig. 1. There was no statistically significant association between the categorical variable < working location > (e.g., university hospital/tertiary care hospital) and the application of neoadjuvant treatment (*p* = 0.68) or the application of TNT (*p* = 0.29). Additionally, there was no statistically significant association between the category of < university hospital/tertiary care hospitals > provider and the remaining institutions regarding the application of TNT (*p* = 0.48). The survey also asked about the recommendations for offering TNT to patients. Multiple response options were provided, including tumor categories (T2, T3, T4), involvement of the mesorectal fascia in MRI (MRF +), nodal status on imaging (N1, N2), and extramural vascular infiltration (EMVI +). The recommendations marked more frequently as "yes" were T3, T4, MRF + , and N2. N1 and EMVI + were roughly equal, while T2 was marked as "no" in 88.9% of cases (compare Fig. 2). Seven participants used the option to add additional information: patient request to avoid surgery, distal rectal cancer, rectal cancer in the middle third, distal T3N0 tumor, patient refusal of surgery, involvement of the lateral lymph nodes in the distal third of the rectum or cT3/c tumor, and involvement of the lateral lymph nodes. In 62 out of 63 cases, tumor board discussion before offering TNT was reported as standard practice. The question regarding the presence of internal written guidelines, such as standard operating procedures (SOP) and clinical pathways (CP), yielded the following responses: 28.6% had internal guidelines, 33.3% had no internal guidelines, 20.6% had guidelines within their department, and 15.9% had clinical pathways across departments. The presence of written guidelines/SOP/CP did not show any association with the category of institutions (*p* = 0.46): four university hospitals/tertiary care hospitals reported having clinical pathways, as did three medical service centers.

#### Questions addressing radiation therapy planning

**Fig. 3:**
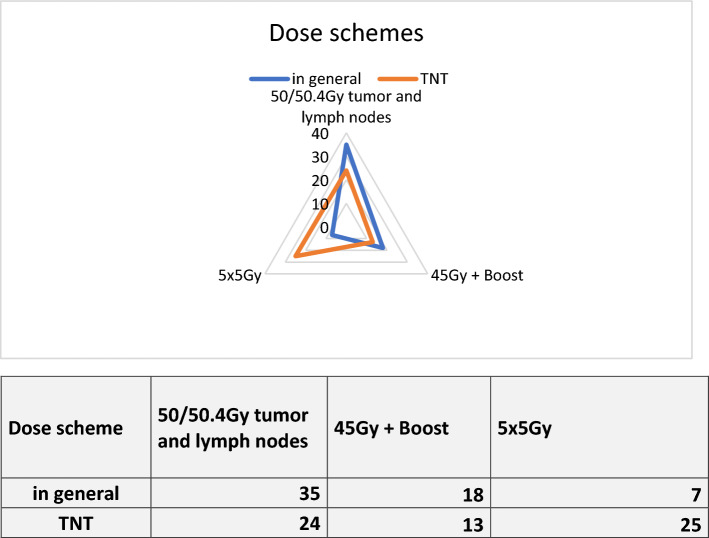
Quoted dose schemes for radiation therapy in general and radiation therapy in TNT, corresponding radar chart

A subsection of 15 questions focused on the radiation therapy planning process. The majority of respondents (77.8%) used planning computed tomography (PLCT) in the supine position, while a smaller portion (19%) used the prone position. Two respondents (3.2%) did not specify their position. None of the participants utilized the option to enter text in a given field. Regarding the use of contrast agents, the largest proportion of respondents (68.3%, *n* = 43) used native PLCT, while 20.6% (*n* = 13) used intravenous contrast and 17.5% (*n* = 11) used orally administered contrast agents. These options were multiple answers, accounting for the possible combination of oral and intravenous contrast agents, resulting in a total slightly above 100%. In terms of radiation therapy planning techniques, the majority of respondents used VMAT (82.5%), followed by IMRT (12.7%), and 3D conformal approaches (3.2%). One participant did not specify their planning technique (1.6%). Survey participants were asked to report their dose scheme for rectal cancer in general and specifically for total neoadjuvant therapy (TNT). Only one answer was possible, with the option to provide a free-text response. The most commonly used scheme for rectal cancer in general was 50/50.4Gy for the tumor and draining lymph nodes. This was followed by 45Gy with an additional boost, and then by 5 × 5Gy scheme. The free-text option was used only twice, with a respondent indicating the use of 5 × 5Gy if the tumor is not located in the lower third of the rectum. Another respondent mentioned a total dose of 45Gy. Dosing schemes for TNT varied slightly, with more participants reporting the use of 5 × 5Gy compared to radiation therapy for rectal cancer in general. The free-text option was used only once, with a respondent specifying the use of 5 × 5Gy if the tumor is not located in the lower third of the rectum. For more detailed information on dosing schemes, refer to Table 2 and Fig. 3. Only 18 out of 63 participants (28.6%) reported treating the inguinal nodes when treating deep-seated rectal cancer. The majority (69.8%, *n* = 44) stated that these nodes are not treated at their institution (missing *n* = 1).

**Table 2:** Information on dosing schemes for treating rectal cancer in general and for TNT

50/50.4Gy tumor and lymph nodes	*n* =	%
In general	35	55.5
TNT	24	38.1
45Gy + Boost		
In general	18	28.6
TNT	13	20.6
5 × 5Gy		
In general	7	11.1
TNT	25	39.7
Missing (both)	In general 2/TNT 1	
Other (in general)	1

Next question addressed whether there are guidelines for treating inguinal nodes. Out of the 42 participants who responded, 24 indicated that they did not have any guidelines, four stated that they do have guidelines, and 14 provided further details. Among those who provided details, one participant mentioned that their decision depended on the results of an MRI, while the rest mentioned factors such as infiltration of the anal canal, cT4 tumor or sphincter infiltration. It is worth noting that out of the 18 participants who mentioned treating inguinal nodes, 29% (13 out of 46) stated that they only treat inguinal nodes in normofractionation, 9.5% (6 out of 63) mentioned treating inguinal nodes with a 5 × 5Gy scheme. We also sought to evaluate whether participants consider an MRI of the pelvis as a requirement for the contouring process. More than two thirds (*n* = 48 out of 63, 76.2%) stated that an MRI is an essential component of the contouring process, while 20.6% (13 out of 63) answered that an MRI is not necessary for contouring. One participant mentioned that an MRI is necessarily for local staging but not specifically for contouring. Two questions focused on generating the clinical target volume (CTV) and the planning target volume (PTV). A total of 81% (51 out of 63) provided answers regarding their CTV/PTV safety margin, while 17.5% (11 out of 63) indicated that they do not create a CTV. The mean safety margin reported was 8.02mm, with a median margin of 8mm (range 5–20 mm, IQR 5–10 mm). The final question regarding the PTV inquired about the inclusion of the sacral foramina. Among the 63 participants who responded, 60.3% (38 out of 63) stated that they include the sacral foramina in the PTV, while 38.1% (24 out of 63) mentioned that they would omit them. Lastly, participants were asked to indicate the organs at risk (OAR) that they typically contour. Almost all participants (98.4%/62 out of 63) contour the bladder, 65.1% contour the bowel bag, 41.3% contour bowel loops, 93.7% contour the femoral heads, and 60.3% contour genitalia.

This question also included a free-text response option, which was utilized by 18 participants. In three cases, the anal sphincter was contoured as an OAR. Other responses included bony sub-structures (sacrum and symphysis), ovary in pre-menopausal women, urethra, penile bulb, and sigmoid colon. The next multiple answer question asked participants to specify specific internal constraints for the bladder, bowel bag, sigma, femoral heads, and others. Details can be found in Table 3. Regarding the questions asking for the application of image-guided radiotherapy (IGRT), participants were allowed to select multiple answers. The majority of survey participants reported using cone-beam CT (CBCT) (*n* = 56, 88.9%). Kilovoltage field control with an additional on-board imaging device was used by 30.2% (*n* = 19) of respondents, while megavoltage image detection with the LINACS energy and image detector was reported by 19% (*n* = 12). Two participants used ExacTrac^®^ and C-Rad^®^ as mentioned in the free-text option. Another free-text question inquired about the weekly frequency of IGRT, and 94% (59/63) of participants provided an answer. Most participants reported using daily CBCT (*n* = 16), while 10 participants replied that they used CBCT 2–3 times a week. Eleven participants reported using an in-house algorithm to determine IGRT frequency, while 11 participants reported using daily kV imaging. Additionally, eleven participants reported using kV imaging 2–3 times a week.

**Table 3:** Dose constraints for given OAR; in (brackets) number of mentions

OAR	Dmax (Gy)	Dmean (Gy)	Vx % or cc	Dx	other
Bladder, *n* = 32	45, 54, 55(2), 56, 60, < 107%Dref, < 65	< 21, 25, 30(2)	**V15** > 15%, < 50% **V21** < 15% **V25** < 5%(2), < 50% **V30** < 60% **V35** < 35%, < 50% **V40** < 10%, < 35%, < 40%(2), < 40%(2), < 50cc, < 70% **V45** < 30%(2), < 45% **V50** < 5%, < 30%, < 45% **V65** < 30%	D05 < 29Gy D10 < 25Gy D50 < 40Gy D25 < 60Gy D12 < 70Gy	ALRA „like study protocol “ • 50.4Gy: V35 < 22%, V50 < 7% • 5 × 5Gy: V21 < 15% V25 < 5% Whole bladder < 50Gy
Bowel bag (n = 24)	30, 45, 50, 51, 51.2, 52, 54(2), < 102%, < 107%	< 17, < 20,	**V10** < 180cc **V12** < 450cc **V16** < 15cc, **V20** < 180cc, < 85cc, **V21** < 20cc **V23** < 85cc **V30** < 450cc(2) **V32** < 150cc **V35** < 150cc(2), < 20% **V40** < 90cc(4), < 200(2) **V45** < 20cc(2), < 170cc, < 195cc, < 250cc		„like study protcol “
Sigmoid colon	26, < 102%, < 54(2), 56(2)			D0.03cc < 57.5	ALARA „like study protcol “ 17% < 65Gy and 40% < 35Gy
Femoral heads	25 if 5 × 5Gy 40, 45(2), < 45, < 46, 50, < 50(4), 56–60,	10Gy 20Gy 21.88Gy < 25Gy < 30-40Gy < 45Gy(2)	**V12** < 11% **V12.5** < 11% if 5 × 5Gy, **V25** < 15% if 50.4Gy **V12.5** < 25% if 5 × 5Gy **V25** < 25% if 50.4Gy **V30** < 50%(2) **V35** < 50% **V40** < 35%(2) **V40** < 5% **V44** < 5% **V50** < 50%	D01 < 36Gy, D10 < 25Gy D2cc < 50Gy	„like study protcol “
Free-text field „others “					Sphincter V21.2 < 60% if 5 × 5Gv,V40 < 40% if 50.4Gy Testes < 18Gy Sphincter V21.2 < 60%, ALARA, Small bowel V45 < 30%, Penile bulb V50 < 95%, Bladder wall Dmean 18.75Gy, „like study protcol “, RTOG, Gonads < 2Gy

#### Questions addressing chemotherapy

**Fig. 4:**
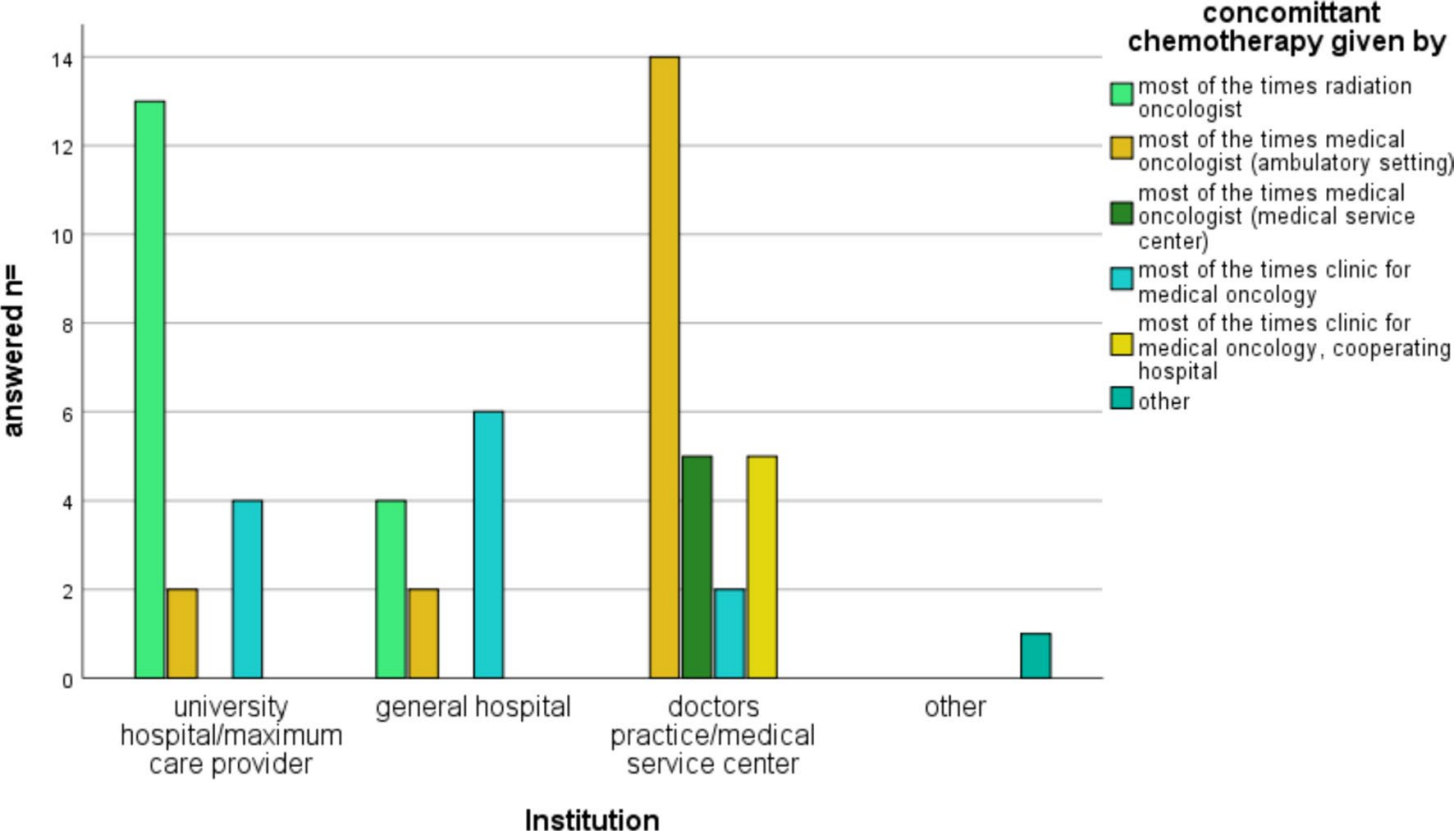
Administration policy of chemotherapy by work setting

The survey contained six questions related to the application, concepts, and administration of chemotherapy. The first question sought to determine who typically administers concomitant chemotherapy during chemoradiotherapy for rectal cancer. Out of the total participants, 16 replied that the radiation oncologist usually administers concomitant chemotherapy, while 18 participants stated that medical oncologists in an ambulatory setting are responsible for its administration. In five cases, chemotherapy is given by a medical oncologist in a cooperating medical service center, and in 12 cases, it is administered by the hospital's clinic for medical oncology. Two participants provided additional information in the free-text option, stating that radiation oncologists always administer the chemotherapy and never medical oncologists, or that it is carried out by both radiation oncologists and medical oncologists in an interdisciplinary chemotherapy unit (“ambulante spezialfachärztliche Versorgung”, ASV). Using a chi-square test, we found a significant association between the variable < institution > which recorded who administer the chemotherapy and the participants’ working setting, as shown in Fig. 4. Generally, larger hospitals (university hospitals/tertiary care hospitals) tend to administer the chemotherapy themselves, while physicians in private practices/medical service centers collaborate with medical oncologists in most cases (*p* < 0.0001).

The next survey question focused on the application of concomitant chemotherapy in TNT concepts, using the same answer options. We observed the same trend as with concomitant chemotherapy in general. Out of 19 participants from university hospitals/tertiary care hospitals, 12 reported that radiation oncologists administer the TNT medication themselves. However, this was only the case in 3 out of 12 general hospitals, and none of the 26 participants working in private practices/medical service centers reported administering the medication themselves (*p* < 0.001). The sequential administration of TNT chemotherapy following radiation therapy varied among the respondents. In two out of 20 cases, the radiation oncology department provided chemotherapy, while in one case it was given by the department of radiation oncology or cooperating partners. In the majority of cases (7/20), the department of medical oncology of the same hospital administered the chemotherapy, and in 6/20 cases, it was provided by a medical oncologist in a private practice. For general hospitals, most of them (8/12 cases) administered sequential chemotherapy in their own department of medical oncology. In 2/12 cases, it was provided via an associated medical service center. In cases where the respondents worked at a doctor's practice or medical service center, the sequential chemotherapy was most commonly given at a medical oncology doctor's practice (14/26 cases). Five cases involved a medical oncologist from an associated medical service center, and six cases involved a department of medical oncology at a cooperative hospital. Statistical analysis showed significant differences in the administration of sequential chemotherapy (*p* < 0.001), with most of the respondents working at medical service centers/doctor’s practice cooperating with medical oncologists in doctor's practice, whereas maximum care providers and general hospitals more often cooperate with a clinic for oncology in their hospital. The next question addressed the use of concurrent chemotherapy regimen during radiation therapy. Multiple options were provided, and the respondents could select more than one medication. The most commonly used options were 5-fluorouracil/orally administered capecitabine, with 64.4% of respondents choosing this medication. Oxaliplatin was used by 37.3%, while irinotecan and folinic acid were chosen by one respondent (1.7%). Few respondents selected FOLFOX4 (6.8%), FOLFOX6 (1.7%), or FOLFIRINOX (3.4%). Four respondents also provided additional comments, with two adhering to the ARO 18.1 protocol, one respondent stating they never used concurrent chemotherapy, and one respondent finding the question provocative and suggesting a preference for participating in a study. The survey also asked about the chemotherapy regimen used after radiation therapy in a TNT protocol. Again, multiple answer options were provided. The majority of respondents chose to administer chemotherapy analogous to the RAPIDO protocol, with 44.1% using mFOLFOX4 and 28.8% using capecitabine/oxaliplatin. Two respondents opted for three courses of FOLFOX (22%), and 18.6% chose 16–18 weeks of mFOLFOX6/CapOx (OPRA-scheme). The FOLFOX6 regimen was selected by 15.3% of respondents. None of the respondents chose FOLFIRINOX for the sequential chemotherapy phase, and 25.4% stated they could not provide a definite answer as they did not administer sequential chemotherapy themselves. Three respondents provided free-text responses, with two mentioning the use of FOLFOX6 according to the ARO 18.1 protocol. A question regarding the duration of sequential chemotherapy was answered by 20 (31.7%) respondents. Among them, 60% administered 4–6 months of sequential chemotherapy, 30% used a duration of up to three months, and in 10% of cases, the duration remained unclear. There was also an option to enter the number of administered courses, which was chosen by 30.2% of respondents. Among those, 10.5% administered up to three courses, 63.2% administered four to six courses, and 26.3% administered more than six courses.

#### Watch and wait strategy

Three questions addressed the topic of a watch and wait (WaW) strategy in this online survey. The first question asked participants if a WaW strategy was implemented in their working environment. Fifty-eight participants responded, and 63.8% of them reported using a WaW strategy, while 36.2% stated they did not. Participants also had the opportunity to describe their specific strategy, which was done by 15.5% of the respondents. In conclusion, the majority of respondents who have a WaW strategy utilize an intensified follow-up scheme every three months, which involves proctoscopy and MRI. When it comes to offering WaW to patients, 41.4% do so actively, while 58.6% do not active[10]ly offer it. In terms of the timepoint at which WaW is offered, the responses were almost evenly split: 50% offer WaW depending on the results of radiochemotherapy, 47% offer it during the informed consent talk, and 3% offer it during neoadjuvant radiation therapy. When asked about the preferred regimen if a WaW strategy is offered right from the beginning of oncological therapy, 62% of the respondents prefer normofractionated TNT, 16% prefer 5 × 5Gy, 19% prefer normofractionation without TNT, and 3% offer WaW only as part of a study protocol.

#### Personnel background

The survey concluded with two personal questions. The first question inquired about the respondent’s years of experience in the field of radiation oncology. The survey primarily attracted an experienced audience, with a median experience of 23 years (95% CI 19.24–23.57). Additionally, 89.7% of these experienced individuals considered TNT to be a game-changer, 6.9% did not share this perspective, and 3.4% expressed uncertainty regarding whether TNT should be recognized as such.

## Discussion

Rectal cancer is a significant global health burden, accounting for a considerable number of cancer-related deaths annually. Over the years, various treatment approaches have been employed in an attempt to improve outcomes and quality of life for patients with rectal cancer. Neoadjuvant therapy has emerged as gold standard in the management local advanced stages of this disease since more than two decades [11–13]. Traditionally, the standard treatment for locally advanced rectal cancer has been a combination of chemotherapy and radiation therapy followed by surgery. However, attempts to achieve WaW have been made since decades [14]. As the management of rectal cancers progresses, new data from randomized controlled trials (such as RAPIDO and PRODIGE 23) have highlighted the importance of total neoadjuvant therapy in treating locally advanced rectal cancer. The findings of TNT studies indicate that total neoadjuvant therapy has demonstrated a notable enhancement in disease-free survival. TNT has been shown to influence the pCR rate and rates of organ preservation [1, 4]. This approach is now considered a standard treatment option for suitable patients. TNT offers the potential for improved control of systemic disease, increased treatment adherence, and reduced time with an ostomy. However, it is worth noting that TNT encompasses various treatment options, varying in terms of radiation dosage, chemotherapy regimen, and sequence of administration. An initial online survey conducted among experts at German Cancer Society (DKG) accredited colorectal cancer centers identified a notable variety in the management of clinical complete response and the implementation of watch and wait strategies [15]. The work addressed TNT treatment with fourteen questions, and the survey was intended to be answered by the center’s colorectal surgeon. The questions dealed with TNT in general, possible study participation of the center, recommendation for TNT and possible concerns regarding TNT treatment. The scope of our survey was to get insight into the TNT application on the radio-oncologic community, which is a keyholder in TNT delivery. For this, we developed a survey with 43 questions in six chapters, which evaluated TNT in a deep way. Even if only data for 63 participants was available for full evaluation, the sample can be regarded as representative, because two conditions were met, as described by Andrade et al.: first of all, study population was known and gathered via professional associations web pages. As second condition, the survey was sent to all institutions, irrespective of university hospital or doctors’ practice [16]. In general, survey response rates are much lower than traditional response rates to telephonic surveys, which in turn are less than response rates to surveys using the face-to-face method. Reasons for this include survey fatigue, competing demands and privacy concerns [17, 18]. The net participation rate of our survey of 21% (82/392 working email adresses) is within the described literature [19]. Unfortunately, 20 participants didn’t deliver more than general information. Nevertheless 63 participants gave detailed information. With roughly 63% of the participants coming from doctors’ practice or general hospitals, participants from university hospitals /tertiary care providers don’t seem to be overrepresented. Nearly all of the colleagues are aware of the consensus opinion on the use of total neoadjuvant therapy (TNT) for rectal cancer. Neoadjuvant treatment for rectal cancer has found its role, with most of the participants treating most of the patients in a neoadjuvant setting, as 76.2% treat > 80% with a neoadjuvant regimen. One third of the respondents treat 21–50% of their patients with TNT, which seems to be in line with the results of the survey among DKG centers, where 35% of the respondents had some concerns about TNT [15]. The missing association between TNT and working location can be seen as a surrogate that TNT has gained broad distribution and acceptance, also out of the academic fields. Fortunately, tumor board discussion of the patient’s case before administering TNT seems to be standard (62/63 cases). Sixtyfive percent answered to have some sort of guideline for TNT, with 15% having a clinical pathway across multiple departments, which seems to be an impressing result three years after the consensus statement. Moreover, nearly 64% have a strategy for WaW. Interestingly, 60% of the respondents of the DKG-survey reported to treat the patients according to RAPIDO protocol. Our results also show, that 5 × 5Gy seems to be used more when applicating TNT in comparison when using standard neoadjuvant radiation therapy (see Table 2). However, when WaW is an initial part of an individualized strategy, 62% of the respondents prefer normofractionation. The results of the multicenter International Watch & Wait Database (IWWD) registry study, which reported on more than 800 patients with a clinical complete response, showed that nearly all patients were treated with normofractionation [20]. Regarding target volume definition for radiation therapy, interestingly only a minority (28.6%) reported to treat inguinal nodes for deep-seated rectal cancer, which, in contrast to the published guidelines [21, 22]. Nevertheless, in the literature, some authors advocate for the omission of treating the inguinal nodes [23]. Interestingly, 60% of 63 respondents reported to include the sacral foramina, which is recommended in the treatment for anal cancer [24], but is not a prerequisite in the treatment of rectal cancer [21]. Given the answers of questions addressing radiation therapy planning, it can be concluded, the high quality IGRT can be seen as standard in German-speaking radiation therapy departments. The subchapter asking for chemotherapy revealed what was anticipated: bigger institutions tend to deliver chemotherapy for themselves, physicians working in doctors’ practice and medical service centers focus on solely on radiation therapy and cooperate with various partners. The pros and cons of both philosophies are beyond the scope of this survey. An experienced audience sees TNT as a game-changer, which is reflected by its broad application with modern IGRT techniques. WaW and organ preservation become more and more a treatment goal of rectal cancer therapy. Possible benefits of an organ-preserving approach range from improved bowel function [10] to better body image, sexual function, and fewer financial problems [25]. The focus on chemotherapy regimens and detailed, radiation oncology-specific questions could be seen as a drawback of our survey. However, at the time the survey was designed, the main discussion and interest of the community was how and with which of the published protocols to implement TNT to improve outcomes. The discussion about the optimal WaW strategy including follow-up programs was still ongoing and should be clarified in these days. Long-term data beyond three years follow-up are available for the OPRA protocol. As shown by others, tumor regrowth after WaW is most common in the first two to three years [20, 26]. Patients treated in the OPRA trial who showed tumor regrowth did so in 99% within three years [27]. We recommend intensified follow-up after TNT, which should be implemented as SOP. Whether the goal of organ preservation will be supported by additional treatment modifications, such as individualized external beam radiotherapy or brachytherapy boost [28], or whether more earlier tumors will be treated with WaW, as in the UK [29], will be shown in the future. Potential limitations of any survey lie in the design of the questions themselves (wording, type of question, e.g., multiple vs. single response). Although this survey provided an indication of the categories that may drive the TNT recommendation (see Fig. 2), more precise and predefined questions on this topic would have provided more clarity for daily practice.

## Conclusion

TNT seems to be implemented in the German-speaking radio-oncologic community. This community delivers modern, image-guided therapy to their patients. Multidisciplinary team decisions and internal guidelines for delivering TNT play an important role. Besides statistically significant differences in the administering policy of chemotherapy, TNT seems to have already changed the rectal cancer treatment regimens toward an organ preservation approach in selected cases. In these WaW cases, normofractionation seems to be preferred over hypofractionation.

## Supplementary Information

Below is the link to the electronic supplementary material.

Supplementary file1 (DOCX 23 KB)

Supplementary file2 (PDF 532 KB)

Supplementary file3 (DOCX 18 KB)

## Data Availability

The datasets of the survey are available from the corresponding author upon reasonable request.
