# Supplementary material for: Assessing the practice of total neoadjuvant therapy for rectal cancer: an online survey among radiation oncology departments in Germany and German-speaking regions of Austria and Switzerland
Source: Clin Exp Med. 2024 Oct 19;24(1):242. doi: 10.1007/s10238-024-01495-w (PMC11490463; doi:10.1007/s10238-024-01495-w)
Supplement: Supplementary file 1 — Supplementary file1 (DOCX 23 KB) [file 10238_2024_1495_MOESM1_ESM.docx]

**Addendum Table 1**

Checklist for Reporting Results of Internet E-Surveys (CHERRIES)

| 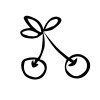 | **Checklist for Reporting Results of Internet E-Surveys (CHERRIES)** according to: Eysenbach G. Improving the quality of Web surveys: the Checklist for Reporting Results of Internet E-Surveys (CHERRIES). J Med Internet Res. 2004 Sep 29;6(3):e34. | |
| --- | --- | --- |
| ***Item Category*** | ***Checklist Item*** | ***Explanation*** |
| **Design** |  |  |
|  | Describe survey design | Radiation oncology departments in Germany, Austria, and German-speaking Switzerland, contacted via publicly available Email addresses. Institutions that are not linked at the DEGRO, ÖGRO or SASRO were not contacted |
| **IRB (Institutional Review Board) approval and informed consent process** |  |  |
|  | IRB approval | IRB approval was obtained from the ethics committee of the University Medical Center Rostock with the reference number A 2022-0183 |
|  | Informed consent | Informed constent given by actively checking a check box by the participant. Estimated time frame was given. Information was given, that data storage is anonymous and in compliance with german DSGVO. Person responsible for content, investigator and data protection officer were given. |
|  | Data protection | Survey is in compliance with German DSGVO, neither IP adresses nor other information like MAC adresses were stored. |
| **Development and pre-testing** |  |  |
|  | Development and testing | Survey was developed using Unipark®, licensed from Medical School Hamburg, usability and technical functionality of the electronic questionnaire had been tested before fielding the questionnaire by SK and MND |
| **Recruitment process and description of the sample having access to the questionnaire** |  |  |
|  | Open survey versus closed survey | “open survey” open for each visitor of the site that got the link or QR code, but selectively distruted |
|  | Contact mode | Publicly available e-mail adresses |
|  | Advertising the survey | No advertisement was done. |
| **Survey administration** |  |  |
|  | Web/E-mail | Link to web page was distributed via e-mail with a corresponding QR code for mobile devices routing tot he web page |
|  | Context | Sample was pre-selected by using only institution for radiation oncology registered at the homepages of DEGRO/ÖGRO/SASRO |
|  | Mandatory/voluntary | It was a voluntary survey |
|  | Incentives | No incentives were offered |
|  | Time/Date | Timeframe were the data was collected was January 22 to April 15, 2023 |
|  | Randomization of items or questionnaires | No randomization was done |
|  | Adaptive questioning | No adaptive questioning (certain items, or only conditionally displayed based on responses to other items) was done |
|  | Number of Items | 43 questions in six sections, each starting with a short introduction |
|  | Number of screens (pages) | Six, with a graphical illustration continously showing the participants progress in the survey. |
|  | Completeness check | Survey could be completed without completing each item |
|  | Review step | Respondents were able to review and change their answers through a Back button before completing the survey |
| **Response rates** |  |  |
|  | Unique site visitor | Due to guarantee anonymity no unique site visitor tracking was done |
|  | View rate (Ratio of unique survey visitors/unique site visitors) | Due to guarantee anonymity no Ratio could be calculated |
|  | Participation rate (Ratio of unique visitors who agreed to participate/unique first survey page visitors) | Due to guarantee anonymity no Ratio could be calculated |
|  | Completion rate (Ratio of users who finished the survey/users who agreed to participate) | 63/93 = 67.7% |
| **Preventing multiple entries from the same individual** |  | Due to guarantee anonymity no unique site visitor tracking was done |
|  | Cookies used | Due to guarantee anonymity no unique site visitor tracking was done |
|  | IP check | Due to guarantee anonymity no unique site visitor tracking was done |
|  | Log file analysis | Due to guarantee anonymity no unique site visitor tracking was done |
|  | Registration | Due to guarantee anonymity no unique site visitor tracking was done |
| **Analysis** |  |  |
|  | Handling of incomplete questionnaires | Fort he analysis of questionnaires with missing items, missing number is given |
|  | Questionnaires submitted with an atypical timestamp | No atypical timestamp was observed |
|  | Statistical correction | No weighting of items has been done |
