# Supplementary material for: Assessing the practice of total neoadjuvant therapy for rectal cancer: an online survey among radiation oncology departments in Germany and German-speaking regions of Austria and Switzerland
Source: Clin Exp Med. 2024 Oct 19;24(1):242. doi: 10.1007/s10238-024-01495-w (PMC11490463; doi:10.1007/s10238-024-01495-w)
Supplement: Supplementary file 2 — Supplementary file2 (PDF 532 KB) [file 10238_2024_1495_MOESM2_ESM.pdf]

Fragebogen

1 Allgemeine Informationen

In was für einer Institution arbeiten Sie?

- ◯ Universitätsklinikum oder Maximalversorger
- ◯ Krankenhaus der Grund- und Regelversorgung
- ◯ Arztpraxis/MVZ
- ◯ Andere (bitte spezifizieren)

Der Träger Ihrer Einrichtung ist:

- ◯ Öffentlich/Kommunal
- ◯ Kirchlich
- ◯ Privat
- ◯ Andere (bitte spezifizieren)

In welchem Land praktizieren Sie?

- ◯ Deutschland
- ◯ Österreich
- ◯ Schweiz
- ◯ Andere (bitte spezifizieren)

Wie viele Bestrahlungsgeräte (Linearbeschleuniger) stehen in Ihrer Abteilung zur Verfügung?

- ◯ 1 - 2
- ◯ 3 - 4
- ◯ 5
- ◯ >5

Wie viele Fachärzte sind in Ihrer Abteilung angestellt?

- ◯ bis 3
- ◯ 4 - 5
- ◯ 6 - 10
- ◯ >10

Wie viele Medizinphysikexperten (MPEs) sind in Ihrer Abteilung angestellt?

- ◯ bis 3
- ◯ 4 - 5
- ◯ 6 - 10
- ◯ >10

Wie viele MTRA sind in Ihrer Abteilung angestellt?

◯ [ ]

Wie viele Patienten werden pro Jahr in Ihrer Abteilung strahlentherapeutisch behandelt?

◯ [ ]

1.1 Standardseite

Vielen Dank! Es folgen nun einige Fragen, die sich auf die total

neoadjuvante Therapie (TNT) beziehen.

2 Strahlentherapie im Rahmen der TNT

Die „Konsentierte Stellungnahme der AIO, der ACO und der ARO zur neoadjuvanten Therapie beim Rektumkarzinom" zur TNT ist mir bekannt.

(http://www.aroonline.de/files/Dateien/AIO-ACO-ARO-Statment%20zu%20TNT%20ASCO%202020_final.pdf)

- ◯ ja
- ◯ nein

Wie viele Patienten mit Rektumkarzinom werden pro Jahr in Ihrer Abteilung behandelt?

- ◯ [ ]

Wie viele hiervon neoadjuvant?

- ◯ bis 20%
- ◯ 21 - 50%
- ◯ 51 - 80%
- ◯ >80%

Wie viele von den neoadjuvant behandelten Patienten werden im Rahmen einer Total Neoadjuvanten Therapie (TNT) behandelt?

- ◯ bis 20%
- ◯ 21 - 50%
- ◯ 51 - 80%
- ◯ >80%

Bei welchen Indikationen bieten Sie eine TNT an?

Mehrfachauswahl möglich

- ☐ T2
- ☐ T3
- ☐ T4
- ☐ MRF+ (mesorektale Faszie im MRT befallen)
- ☐ N1
- ☐ N2
- ☐ EMVI+ (Vorhandensein einer extramuralen vaskulären Infiltration)
- ☐ weitere:

Erfolgt regelmäßig eine Vorstellung im interdisziplinären Tumorboard VOR Einleitung TNT?

- ◯ ja
- ◯ nein

Bieten Sie die TNT gezielt zum organerhaltenden Vorgehen an?

- ◯ ja
- ◯ nein

Gibt es in Ihrer Abteilung interne schriftliche Richtlinien/SOPs/Clinical Pathways darüber, bei welchen Indikationen und wie die TNT durchgeführt wird?

- ◯ ja
- ◯ nein
- ◯ SOP abteilungsintern
- ◯ Clinical Pathway abteilungsübergreifend

2.1 Standardseite

Vielen Dank! Es folgen nun einige Fragen zur Strahlentherapieplanung.

3 Bestrahlungsplanunsplanung und -durchführung

Welche CTs fahren Sie zur Bestrahlungsplanung am häufigsten?

- ◯ Rückenlage
- ◯ Bauchlage/“Dünndarmfalle“
- ◯ Andere (bitte spezifizieren):

Wenden Sie bei der Durchführung des Planungs-CTs Kontrastmittel (KM) an?

Mehrfachauswahl möglich

- ☐ oral
- ☐ intravenös
- ☐ kein KM

Welche Verfahren kommen bei der Strahlentherapieplanung am häufigsten zur Anwendung?

- ◯ Bestrahlung mit IMRT
- ◯ Bestrahlung mit VMAT
- ◯ 3D konformale Bestrahlung
- ◯ Vierfelderbox-Planung
- ◯ Weitere:

Was ist Ihr bevorzugtes (am häufigsten genutztes) Dosiskonzept bei der neoadjuvanten Strahlentherapie des Rektumkarzinoms allgemein?

- ○ 50/50.4Gy Primärtumor und Lymphabflusswege
- ○ 45Gy + Boost
- ○ 45Gy
- ○ 5x5Gy
- ○ Andere (bitte spezifizieren)

Was ist Ihr bevorzugtes (am häufigsten genutztes) Dosiskonzept bei der neoadjuvanten Strahlentherapie des Rektumkarzinoms im Rahmen der TNT?

- ○ Andere (bitte spezifizieren)
- ○ 50/50.4Gy Primärtumor und Lymphabflusswege
- ○ 45Gy + Boost
- ○ 45Gy
- ○ 5x5Gy

Behandeln Sie bei tief sitzenden Rektumkarzinomen die inguinalen Lymphabflusswege?

- ○ ja
- ○ nein

Falls Sie bei tief sitzenden Rektumkarzinomen die inguinalen Lymphabflusswege behandeln, gibt es eine klare interne Richtlinie wann?

- ○ ja
- ○ nein
- ○ falls ja, Angabe (z.B. Zentimeter ab ano, T4...)

Falls Sie bei tief sitzenden Rektumkarzinomen die inguinalen Lymphabflusswege behandeln, im Rahmen welchen Dosierungskonzeptes?

Mehrfachauswahl möglich

- ☐ Normofraktionierung
- ☐ auch im Rahmen einer Hypofraktionierung bis 3Gy
- ☐ auch im Rahmen von 5x5Gy

Ist bei Ihnen das Vorhandensein eines MRT des Beckens Voraussetzung zur Konturierung?

- ◯ ja
- ◯ nein
- ◯ andere/Freitext

Wie groß ist Ihr Sicherheitssaum CTV – PTV in Zentimetern?

- ◯ Konturieren kein CTV
- ◯ Saum in cm:

Schließen Sie die sakralen Foramina in das CTV ein?

- ◯ ja
- ◯ nein

Welche Risikostrukturen konturieren Sie bei der Bestrahlung von Patienten mit Rektumkarzinom standardmäßig?

Mehrfachauswahl möglich

- ☐ Blase
- ☐ Bowel Bag
- ☐ Einzelne Darmschlingen
- ☐ Hüftköpfe
- ☐ Genitalia
- ☐ andere Strukturen (bitte spezifizieren):

Haben Sie abteilungsinterne Dosisvorgaben (Constraints, Dmax, D1% o.Ä.) für:

(bitte Angabe, z.B. Dmax Blase 56Gy oder Bowel Bag 200ccm <40Gy)

- [ ] Blase:
- [ ] Bowel Bag:
- [ ] Sigma:
- [ ] Hüftköpfe:
- [ ] Andere:

Welche IGRT Bildgebung nutzen Sie?

(Mehrfachauswahl möglich)

- [ ] CBCT (KV oder MV)
- [ ] KV-Aufnahmen (zusätzlicher On-Board Imager)
- [ ] MV-Aufnahmen (MV Energie des Beschleunigers und Detektor)
- [ ] weiteres:

In welcher Häufigkeit wenden Sie IGRT an?

Freitext, z.B. 2x/Woche MV-Aufnahmen, tgl. CBCT usw.

- [ ] Freitext:

3.1 Einleitung Chemotherapie

Vielen Dank! Es folgen nun einige Fragen zur Chemotherapie.

4 Chemotherapie und Durchführung

Wer appliziert die parallele Chemotherapie bei kombinierten Radiochemotherapien des Rektumkarzinoms allgemein (ohne TNT)?

- ◯ Meistens Radioonkologe/Strahlentherapeut
- ◯ Meistens ein Onkologe (Niedergelassen/Kooperationspraxis)
- ◯ Meistens ein Onkologe (zugehöriges MVZ)
- ◯ Meistens die Klinik für Onkologie im Hause
- ◯ Meistens eine Klinik für Onkologie (Kooperationskrankenhaus)
- ◯ Teils Radioonkologe/teils onkologische Partner
- ◯ Andere (bitte spezifizieren)

Wer appliziert die parallele Chemotherapie im Rahmen der TNT?

- ◯ Meistens Radioonkologe/Strahlentherapeut
- ◯ Meistens ein Onkologe (Niedergelassen/Kooperationspraxis)
- ◯ Meistens ein Onkologe (zugehöriges MVZ)
- ◯ Meistens die Klinik für Onkologie im Hause
- ◯ Meistens eine Klinik für Onkologie (Kooperationskrankenhaus)
- ◯ Teils Radioonkologe/teils onkologische Partner
- ◯ keine parallele Chemotherapie, da 5x5 Gy
- ◯ Andere (bitte spezifizieren)

Wer appliziert die sequentielle Chemotherapie (nach abgeschlossener Strahlentherapie) im Rahmen der TNT?

- ◯ Meistens Radioonkologe/Strahlentherapeut
- ◯ Meistens ein Onkologe (Niedergelassen/Kooperationspraxis)
- ◯ Meistens ein Onkologe (zugehöriges MVZ)
- ◯ Meistens die Klinik für Onkologie im Hause
- ◯ Meistens eine Klinik für Onkologie (Kooperationskrankenhaus)
- ◯ Teils Radioonkologe/teils onkologische Partner
- ◯ Andere (bitte spezifizieren)

Welche Substanzen/Protokolle werden parallel zur Strahlentherapie im Rahmen der TNT appliziert?

Mehrfachnennung möglich

- ☐ 5-Fluoruracil
- ☐ Capecitabin
- ☐ Oxaliplatin
- ☐ Irinotecan
- ☐ Folinsäure
- ☐ FOLFOX4
- ☐ FOLFOX6
- ☐ FOLFIRINOX
- ☐ Andere (bitte spezifizieren)

Welche Substanzen werden nach der Strahlentherapie im Rahmen der TNT appliziert?

Mehrfachnennung möglich

- ☐ mehrere Kurse CapOx nach Rapido
- ☐ mehrere Kurse mFOLFOX4 nach Rapido
- ☐ drei Kurse FOLFOX (nach CAO/ARO/AIO-16-Studie)
- ☐ 16 - 18 Wochen mFOLFOX6/CapOx nach OPRA
- ☐ FOLFOX6
- ☐ FOLFIRINOX
- ☐ wir applizieren die Chemotherapie nicht selber, bin mir nicht sicher
- ☐ Andere (bitte spezifizieren)

Wie lange wird die Chemotherapie nach der Strahlentherapie durchgeführt (sequentielle Phase):

(Mehrfachauswahl möglich)

- ☐ wir applizieren die Chemotherapie nicht selber, bin mir nicht sicher
- ☐ Dauer (Monate)
- ☐ Anzahl Kurse

5 Einleitung W%W

Vielen Dank! Es folgen nun einige Fragen zur Watch and Wait Strategie.

6 watch and wait

Haben Sie an Ihrer Einrichtung ein „Watch&Wait“ Konzept implementiert, falls sich nach der TNT vor der geplanten OP eine Komplettremission zeigen sollte?

- ja
- nein
- falls ja, Konzept skizzieren:

Empfehlen Sie Ihren Patienten „Watch&Wait“ aktiv?

- ja
- nein

Falls Sie Ihren Patienten „Watch&Wait“ aktiv empfehlen, wann?

- schon vor der Therapie/beim Aufklärungsgespräch
- während der laufenden Strahlentherapie
- abhängig vom Ergebnis der Strahlentherapie (überprüft z. B. durch erneute Endoskopie)

Falls Sie Ihren Patienten „Watch&Wait“ von Beginn an empfehlen, verschreiben Sie dann bevorzugt

- die TNT in Normofraktionierung
- die TNT mit 5x5Gy
- konventionell fraktioniert 45-50/50.4Gy ohne TNT
- Andere (bitte spezifizieren)

6.1 Persönliches

Vielen Dank! Es folgen nun noch 2 Fragen zu Ihrem Hintergrund

7 Persönliches

Seit wievielen Jahren sind Sie in der Strahlentherapie tätig?

- ◯ Ich bin (Anzahl Jahre) tätig:

Denken Sie, dass die bisherigen Studienergebnisse zur TNT die Therapie des Rektumkarzinoms nachhaltig verändert haben?

- ◯ Ja
- ◯ Nein
- ◯ Weiß nicht

8 Endseite
