# Supplementary material for: Assessing the practice of total neoadjuvant therapy for rectal cancer: an online survey among radiation oncology departments in Germany and German-speaking regions of Austria and Switzerland
Source: Clin Exp Med. 2024 Oct 19;24(1):242. doi: 10.1007/s10238-024-01495-w (PMC11490463; doi:10.1007/s10238-024-01495-w)
Supplement: Supplementary file 3 — Supplementary file3 (DOCX 18 KB) [file 10238_2024_1495_MOESM3_ESM.docx]

| Kind of Institution | Germany | Austria | Switzerland |
| --- | --- | --- | --- |
| University clinic/Tertiary care hospital | 16 | 2 | 4 |
| General Hospital | 7 | 3 | 2 |
| Private Practice/Medical Cervice Center | 27 | 1 | 1 |
| Institutional Sponsorship |  |  |  |
| Public | 15 | 5 | 5 |
| Church funded | 7 | 0 | 0 |
| Private | 29 | 0 | 2 |
| Number of LINACS |  |  |  |
| 1 – 2 | 34 | 1 | 4 |
| 3 – 4 | 12 | 2 | 3 |
| 5 | 4 | 1 | 0 |
| >5 | 1 | 1 | 0 |
| Existance SOP/Clinical Pathway |  |  |  |
| yes | 17 | 0 | 1 |
| no | 16 | 5 | 21 |
| SOP internal use | 11 | 0 | 2 |
| Clinical Pathway for Center | 6 | 0 | 4 |
| Usage of TNT for upfront intended WaW |  |  |  |
| yes | 36 | 3 | 7 |
| no | 13 | 2 | 0 |
| WaW strategy in case of clinical complete remission |  |  |  |
| Yes | 23 | 2 | 3 |
| No | 15 | 3 | 2 |
| Strategy Description given by Participant | 8 | 0 | 1 |

Table 4: descriptive statistics on different information grouped by country
